# The burden of influenza among Kenyan pregnant and postpartum women and their infants, 2015–2020

**DOI:** 10.1111/irv.12950

**Published:** 2022-01-23

**Authors:** Nancy A. Otieno, Bryan O. Nyawanda, Meredith McMorrow, Martina Oneko, Daniel Omollo, Shirley Lidechi, Marc‐Alain Widdowson, Brendan Flannery, Sandra S. Chaves, Eduardo Azziz‐Baumgartner, Gideon O. Emukule

**Affiliations:** ^1^ Kenya Medical Research Institute Center for Global Health Research Kisumu Kenya; ^2^ Centers for Disease Control and Prevention, National Center for Immunization and Respiratory Diseases Influenza Division Atlanta Georgia USA; ^3^ Centers for Disease Control and Prevention Division of Global Health Protection Nairobi Kenya; ^4^ Institute of Tropical Medicine Antwerp Belgium; ^5^ Centers for Disease Control and Prevention, Influenza Program Nairobi Kenya

**Keywords:** burden, infants, influenza, pregnant women

## Abstract

**Background:**

In tropical Africa, data about influenza‐associated illness burden are needed to assess potential benefits of influenza vaccination among pregnant women. We estimated the incidence of influenza among pregnant women and their infants in Siaya County, Kenya.

**Methods:**

We enrolled women at <31 weeks of gestation and conducted weekly follow‐up until 6‐month postpartum to identify acute respiratory illnesses (ARIs). We defined ARI among mothers as reported cough, rhinorrhoea or sore throat and among infants as maternal‐reported cough, difficulty breathing, rhinorrhoea or clinician diagnosis of respiratory illness. We collected nasal/nasopharyngeal and oropharyngeal swabs from mothers/infants with ARI and tested for influenza A and B using molecular assays. We calculated antenatal incidence of laboratory‐confirmed influenza among mothers and postnatal incidence among mothers and infants.

**Results:**

During June 2015 to May 2020, we analysed data from 3,026 pregnant women at a median gestational age of 16 weeks (interquartile range [IQR], 13, 18) and followed 2,550 infants. Incidence of laboratory‐confirmed influenza during pregnancy (10.3 episodes per 1,000 person‐months [95% confidence interval {CI} 8.6–11.8]) was twofold higher than in the postpartum period (4.0 [95% CI 2.6–5.5]; *p* < 0.01). Incidence was significantly higher among human immunodeficiency virus (HIV)‐infected pregnant women (15.6 [95% CI 11.0–20.6] vs. 9.1 [95% CI 7.5–10.8]; *p* < 0.01). Incidence among young infants was 4.4 (95% CI 3.0–5.9) and similar among HIV‐exposed and HIV‐unexposed infants.

**Conclusion:**

Our findings suggest a substantial burden of influenza illnesses during pregnancy, with a higher burden among HIV‐infected mothers. Kenyan authorities should consider the value of vaccinating pregnant women, especially if HIV infected.

## 1 INTRODUCTION

Pregnant women have been observed to frequently seek medical care for influenza‐associated illnesses and are at high risk of hospitalization.^1^, ^2^, ^3^ Studies documented an increased burden of influenza among pregnant women during the 2009 influenza pandemic.^4^ However, the few studies reporting seasonal influenza incidence among pregnant women and their infants are typically neither from tropical nor low‐ and middle‐income countries (LMICs).^5^ Although studies from temperate regions suggest that pregnant women with comorbidities^6^, ^7^ and those in the second and third trimesters have higher risk of influenza‐associated hospitalization compared with nonpregnant women,^1^, ^8^ pregnant women in LMICs may be disproportionately affected by influenza infections.^9^ Many tropical areas of Africa have a high prevalence of comorbidities like human immunodeficiency virus (HIV), tuberculosis (TB) and malaria^10^, ^11^ that may influence both incidence and complications of influenza illness.^12^, ^13^ Moreover, year‐round circulation of influenza viruses^14^ may contribute to risk of influenza during pregnancy^7^ in ways that might differ from countries with distinct seasonality.

In 2012, the World Health Organization (WHO) recommended prioritization of influenza vaccination among pregnant women in countries initiating or expanding influenza immunization programmes.^15^ The recommendation was based on increased risk of severe influenza illness and hospitalization observed among pregnant women during seasonal influenza epidemics and the 2009 pandemic^16^, ^17^ and the safety and effectiveness of influenza vaccines during pregnancy.^18^, ^19^ WHO also highlighted the potential benefits of maternal vaccination for infants.^20^ Children born to mothers vaccinated during pregnancy receive passive protection against influenza during their first months of life, when influenza vaccination is not recommended for infants.^21^, ^22^ Despite the WHO recommendation, few LMICs routinely vaccinate pregnant women against influenza, in part because of limited data about the burden of disease in this group.^23^

Kenya, like most middle‐income countries, has yet to adopt the WHO recommendation for maternal influenza vaccination and currently has no national influenza vaccination programme for any target group. In 2016, Kenya's National Immunization Technical Advisory Group provisionally recommended influenza vaccination among children aged 6–23 months;^24^ recommendations for maternal vaccination were deferred until data about influenza burden during pregnancy were available. Here, we estimate the incidence of medically attended influenza illness among Kenyan pregnant women and their infants to inform policy recommendations.

## 2 MATERIALS AND METHODS

### 2.1 Study population

In 2015, the Kenya Medical Research Institute (KEMRI) in collaboration with the U.S. Centers for Disease Control and Prevention (CDC) initiated a prospective cohort study to estimate the burden of influenza disease in pregnancy and its impact on birth outcomes in western Kenya. The study was conducted in two public hospitals in Siaya County: Siaya County Referral Hospital and Bondo sub‐County Hospital. Siaya County has one of Kenya's highest burdens of maternal and infant mortality;^25^ in 2014, maternal mortality was 692 per 100,000 (compared with the national average of 362 per 100,000) and infant mortality was 50 per 1,000 live births (national average, 39 per 1,000).^26^ Among women aged 15–49 years, HIV prevalence was 22%, compared with the national average of 5%.^27^ Malaria is endemic in the region with an overall prevalence of 37%,^28^ and routine surveillance suggests that approximately 20% of pregnant women test positive for malaria during their first antenatal care visit (unpublished KEMRI data). An earlier study in the same region documented 18% parasite prevalence among pregnant women attending first antenatal care visit.^29^

### 2.2 Recruitment and eligibility

Study participants were recruited during home visits or when they came to study hospitals for routine antenatal care visits. For home‐based enrolment, trained community health volunteers (CHVs) administered rapid urine pregnancy tests at home and referred women who tested positive for human chorionic gonadotropin hormone to the nearest study facility for further screening.

Pregnant women were eligible for participation if they (i) were under 31 weeks of gestation (established by ultrasound or fundal height), (ii) aged 15–49 years, (iii) resided within 10 km of study facilities and planned to remain in the study area for the next 12 months, (iv) consented to HIV counselling and testing, (v) agreed to all follow‐up visits, and (vi) were willing to deliver at the study facility. Enrolment was restricted to consenting, women with singleton pregnancies (because of likelihood of delivering low birth weight babies among women with twin pregnancies) and those without leg or spinal deformity or history of fistula repair (because of risks for complicated delivery).

### 2.3 Participant characteristics

Before enrolment, study staff obtained written informed consent from women who were willing to participate in the study. Nurses and clinical officers then obtained detailed medical and obstetric histories from enrolled participants and provided HIV counselling and rapid diagnostic testing. The staff also collected demographics, participant contact information for follow‐up, current and past smoking history, use of medications such as anti‐malarial medication (including sulfadoxine–pyrimethamine for intermittent preventive treatment in pregnancy) and other medications, and hospitalizations during the current pregnancy. Health screening included vital signs, physical and obstetric examination (fundal height and presentation, fetal heart sounds and vaginal examination). Mothers were screened for high blood pressure and oedema to evaluate risk of pregnancy‐induced hypertension or preeclampsia. Laboratory examinations included blood grouping (ABO and Rhesus factor), haemoglobin count, urinalysis, venereal disease research laboratory tests, blood smear examination and rapid diagnostic tests for malaria. Medical history included history of TB or active TB on treatment, asthma, chronic obstructive pulmonary disease (COPD)/chronic bronchitis, diabetes, hypertension, epilepsy or other chronic illness. For HIV‐infected women, we documented information about the use of highly active antiretroviral therapy (HAART) and daily use of cotrimoxazole.

### 2.4 Surveillance for ARIs

We conducted weekly surveillance among participants and their infants to identify acute respiratory illnesses (ARIs), using participants' preferred contact method (telephone or home visits by CHV or study staff), until delivery and for 24‐week postpartum for both mother and infant. Participants not reachable after three consecutive weeks were declared lost to follow‐up at the time of last contact. We defined ARIs among pregnant and postpartum mothers as reported cough, runny nose or sore throat and among infants as reported cough, difficulty breathing, runny nose or clinician‐diagnosed respiratory illnesses in the past 7 days, during weekly follow up, and within the past 10 days during clinic visits for mothers and infants.

We also inquired about any non‐respiratory illnesses among pregnant women, mothers and infants. Patients were tested for malaria if they were symptomatic or if clinicians/nurses suspected malaria infection. Mothers nearing their expected date of delivery were reminded to deliver at study hospitals. At delivery, study staff examined mothers and newborns and documented birth outcomes. For deliveries that occurred outside study facilities, maternal and newborn examinations were conducted within 72 h of birth.

Mothers reporting ARI symptoms or symptoms in their infants during weekly surveillance were asked to come to study clinics for further assessment and management by a study clinician. Respiratory specimens (nasopharyngeal [NP]/nasal [NS] and oropharyngeal [OP]/throat swabs) were collected at study clinics from mothers or/and infants who met criteria for ARI. As a benefit of participation, additional specimens including blood smear for malaria tests, and urine for urinalysis was collected for diagnostics and patient management as per Ministry of Health guidelines. Episodes occurring 14 or more days after a previous ARI episode were considered new episodes.

### 2.5 Laboratory methods

Swabs collected (NS/NP and OP) from a patient presenting with ARI were combined and kept between 2°C and 8°C in cryovials with viral transport medium at field sites, then transported at the end of the day to KEMRI central laboratories located at the main field station in Kisian–Kisumu for testing. The respiratory specimens were tested with real‐time reverse transcription polymerase chain reaction (RT‐PCR) for influenza A and B viruses, using primers and probes provided by CDC's Influenza Division, USA.^30^ Assays were considered positive at cycle thresholds of <40.0.

### 2.6 Data analysis

We describe demographic and clinical characteristics of pregnant women and pregnancy outcomes, including their infants using counts, percentages, medians, ranges and interquartile ranges (IQRs). We defined the gestational trimesters as first, at ≤13 weeks; second, from 14 to 27 weeks; and third, ≥28 weeks. Periods of increased influenza activity during the year were marked out as months where more specimens were testing positive for influenza than the national average of 7%.

We calculated influenza incidence per 1,000 person‐months stratified by maternal HIV status or presence of any chronic condition. Chronic conditions were defined as asthma, TB, diabetes, COPD/chronic bronchitis, epilepsy or hypertension (including essential or pregnancy‐induced hypertension and preeclampsia/eclampsia). We had no access to HIV testing data for infants; we therefore stratified our analysis by HIV exposure for the infants.

In calculating rates, we divided the number of laboratory‐confirmed influenza episodes by person‐months of follow‐up and adjusted for the proportion of ARI episodes for which specimens were not tested and for episodes reported during weekly follow‐up but not sampled. To estimate the 95% uncertainty interval (confidence interval [CI]), we ran 1,000 iterations allowing for the proportion sampled (tested) and the proportion that tested positive for influenza to vary assuming a binomial distribution defined by the actual observed proportions. We used the 2.5th and 97.5th values to estimate the lower and upper limits of the 95% CI. Antenatal person‐time was calculated from enrolment to pregnancy outcome (delivery or miscarriage), maternal death or loss to follow‐up; postpartum person‐time was calculated from delivery to maternal death, loss to follow‐up or study completion at 24 weeks for mothers, and postnatal person‐time from birth to infant death, loss to follow‐up or study completion at 24 weeks for infants. We performed analyses using Stata version 13.0 (Stata Corp., College Station, TX). Statistical significance was considered at *p* < 0.05.

## 3 RESULTS

### 3.1 Enrolment, demographics and maternal person‐time

**FIGURE 1:**
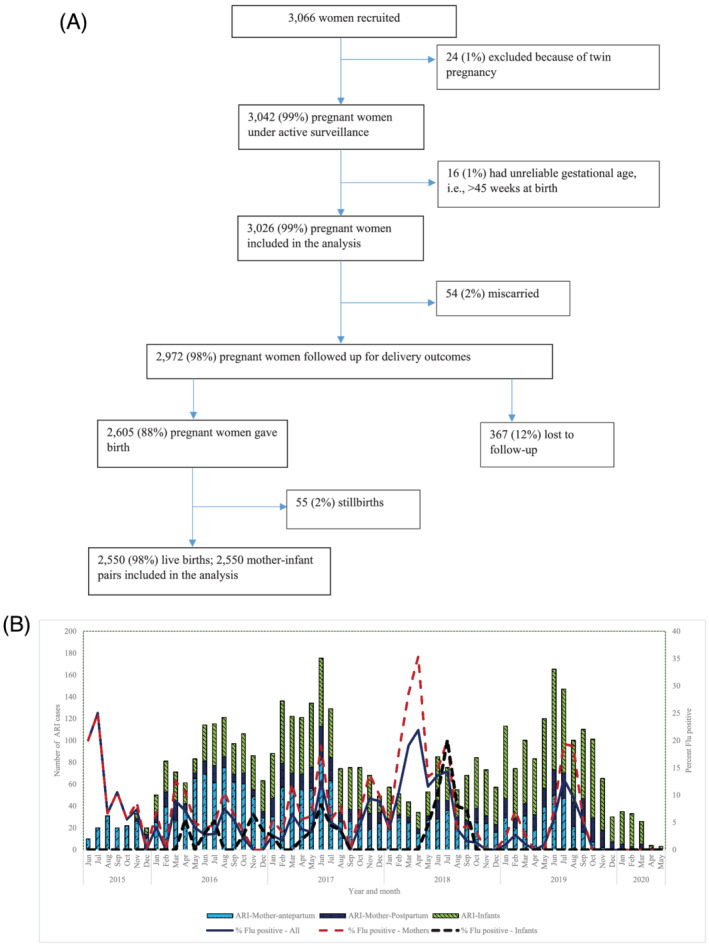
(A) Flow chart of recruitment and follow‐up of study participants. (B) Distribution of acute respiratory illness (ARI) and influenza by month and year

From June 2015 through May 2019, we screened 3,217 pregnant women, enrolled 3,066 who met the criteria for study participation and included 3,026 in our analyses (Figure 1A); 24 mothers who gave birth to twins and 16 mothers with implausible dates of conception (>45 weeks at the time of delivery) were excluded. A total of 2,902 (95.9%) mothers received weekly telephone follow‐up, 70 (2.3%) received visits from CHVs, and 54 (1.8%) were contacted by study staff in person. The median age (IQR) for women and gestational age at enrolment was 24.5 (21.1, 29.0) years and 16 (13, 18) weeks, respectively (Table 1). Half of the women (53.1%) were aged less than 25 years, and most (2,145, 70.9%) were enrolled during their second trimester of pregnancy. Women contributed 15,479 and 9,360 total person‐months during pregnancy and postpartum periods, respectively. The median (IQR) person‐time contributed during pregnancy and postpartum period was 5.1 (4.1, 5.9) and 2.8 (2.8, 2.9) person‐months, respectively.

**TABLE 1:** Demographic and clinical characteristics of pregnant women enrolled in the maternal–infant study in Kenya, June 2015 to May 2020, *N* = 3,026

Characteristic	*n*	%
Age at enrolment, median (IQR)	24.5	(21.1, 29.0)
15–24 years	1,608	53.1
25–34 years	1,235	40.8
35–49 years	183	6.0
Parity, median (range)	1	(0, 11)
First pregnancy		
Yes	806	26.6
No	2,220	73.4
Gestational age at enrolment, median (IQR)	16	(13, 18)
First trimester	873	28.9
Second trimester	2,145	70.9
Third trimester	8	0.3
Comorbidities during pregnancy		
Any chronic condition	647	21.4
Respiratory illness‐related		
Asthma	74	2.5
COPD	4	0.1
HIV	557	18.4
On HAART	534	95.9
On Cotrimoxazole	529	95.0
TB or on TB treatment	14	0.5
Diabetes	6	0.2
Hypertension	18	0.6
Epilepsy	8	0.3
Malaria during pregnancy	1,050	34.7
One episode	818	27.0
>1 episode	232	7.7
At enrolment (first antenatal care visit)	661	21.8
Follow‐up	565	18.7
No. of participants by ARI episodes	1,250	41.3
1 episode	850	28.1
2 episodes	271	9.0
≥3 episodes	129	4.3
Any hospitalization during pregnancy	188	6.2
Hospitalized with ARI	8	0.2
Length of stay^a^		
1–3 days	5	0.2
≥4 days	2	0.1
Hospitalized and influenza positive	0	—

Abbreviations: ARI, acute respiratory illness; COPD, chronic obstructive pulmonary disease; HAART, highly active antiretroviral therapy; HIV, human immunodeficiency virus; IQR, interquartile ranges; TB, tuberculosis.

^a^ One patient left the hospital prematurely before discharge.

### 3.2 Comorbid conditions among pregnant women

Among 3,026 pregnant women, 557 (18.4%) tested positive for HIV or had documented HIV infection (Table 1). Nearly all the women with HIV were receiving HAART (95.9%) and cotrimoxazole prophylaxis (95.0%). Seventy‐four (2.5%) women had asthma, and four (0.1%) had COPD. Eighteen (0.6%) were hypertensive, six (0.2%) diabetic, and eight (0.3%) epileptic. Fourteen (0.5%) women had TB or were receiving current TB treatment. All women were tested for malaria at enrolment and during sick visits, and malaria parasitaemia was identified in 1050 (34.7%).

### 3.3 Description of pregnancy and birth outcomes and infant follow‐up

Among the 3,026 participating women, 54 (1.8%) had miscarriages prior to 20‐week pregnancy, 2,605 (86.1%) gave birth, and 367 (12.1%) were lost to follow‐up (Figure 1A). Of those that gave birth, 55 (2.1%) had stillbirths at a median (IQR) gestational age of 37.5 (32, 39) weeks, and the remaining 2,550 (97.9%) bore live term babies at a median (IQR) gestational age of 39 (37, 40) weeks. All the 2,550 live infants were included in the study. Five maternal deaths occurred postpartum: Three of these deaths occurred within 14 days after delivery (Table S1). No deaths occurred during pregnancy. None of the deaths were associated with respiratory illness. When we assessed the characteristics of women who were lost to follow‐up compared with those who completed the study, younger women <25 years old (63.5% vs. 51.9%, *p* < 0.01) and those in their first pregnancies (40.1% vs. 24.8%, *p* < 0.01) were more likely to be lost to follow‐up (Table S2).

The mean birth weight for the live infants was 3,226 g (SD, 479.3); 96.4% were within normal weight for their gestational age (*z*‐score > −2.0), 1.8% had low birth weight (−3.0 < *z*‐score < −2.0), and 1.6% had very low birth weight (*z*‐score < −3.0) (Table 2). Fifty‐one percent were male (1,300/2,550). Twenty‐three infants (23/2,550, 1.0%) died, and most of these deaths (*n* = 20, 87.0%) occurred within the first 14 days of life. Nineteen (82.6%) deaths were attributed to birth asphyxia, 2 (8.6%) to pneumonia, one (4.3%) to neonatal sepsis, and one (4.3%) to congenital malformation.

**TABLE 2:** Characteristics of infants born in the maternal–infant study in Kenya, June 2015 to May 2020, *N* = 2,550

Characteristic	*n*	%
Sex		
Male	1,300	51.0
Female	1,250	49.0
Prematurity		
<37 weeks	396	15.5
<32 weeks	23	0.9
<28 weeks	4	0.2
Birth weight, mean (SD)	3,225.7	(479.3)
Normal (*z*‐score > − 2.0)	2,464	96.4
Low (−3.0 < *z*‐score < −2.0)	45	1.8
Very low (*z*‐score < −3.0)	40	1.6
5‐min apgar score, <7	32	1.3
Respiratory distress syndrome	31	1.2
Small for gestational age	22	0.9
Perinatal jaundice	3	0.1
Ballard score, mean (SD)	39.2	(3.1)
Birth anomaly^a^	235	9.2
No. of infants by ARI episodes	1,256	49.3
1 episode	744	29.2
2 episodes	330	12.9
≥3 episodes	182	7.1
Other co‐infections in infancy		
HIV exposed	496	19.5
Malaria	142	5.6
TB (exposed)	11	0.4
Any hospitalization	32	1.3
Respiratory illness–related	15	0.6
Hospitalized and influenza positive	0	—
Infant death	23	1.0
<14 days	20	87.0
14–27 days	1	4.4
28 days to 12 weeks	2	8.7

Abbreviations: ARI, acute respiratory illness; HIV, human immunodeficiency virus; SD, standard deviation; TB, tuberculosis.

^a^ Most of the anomalies were minor including extra digits, diastasis recti, umbilical hernia, etc.

### 3.4 ARI and influenza among women

During the study period, our surveillance identified 1,828 ARI episodes among 1,250 women during pregnancy; 400 (32.0%) women had >1 illness episode, and 129 (10.3%) had ≥3 episodes (Table 1). Specimens were collected for 1,613 (88.2%) ARI episodes and were tested for influenza A and B; 140 (8.7%) tested positive. During this period, 188 (6.2%) of 3,026 women were hospitalized for any illness during pregnancy, including eight (4.3%) with respiratory illnesses; none of the pregnant women hospitalized with ARI tested positive for influenza. The study identified 625 ARI episodes in the postpartum period among 501 women; 503 (80.5%) specimens were tested for influenza A and B, and 30 (6.0%) tested positive. Ninety‐five (29.0%) women had ≥1 illness episode, and 23 (5%) had ≥3 episodes. Sixteen postpartum women were hospitalized for any illness, and none had a respiratory illness.

### 3.5 Incidence rates of influenza among women

The incidence of influenza during pregnancy was 10.3 (95% CI 8.6–11.8) per 1,000 person‐months (12.3 [95% CI 10.3–14.2] per 100 person‐years) (Table 3A). Incidence was twofold higher during pregnancy (10.3 [95% CI 8.6–11.8] per 1,000 person‐months) compared to the postpartum period (4.0 [95% CI 2.6–5.5]; *p* < 0.01). The incidence was 3.3 (95% CI 1.6–5.3) and 4.5 (95% CI 2.7–6.4) per 1,000 person‐months during the puerperium and >6‐week postpartum period, respectively. Antenatal rates during each trimester were similar (11.8, 9.0 and 11.6 per 1,000 person‐months in the first, second and third trimester, respectively). Rates of influenza illness among HIV‐infected pregnant women (15.6 [95% CI 11.0–20.6]) were nearly twice those among HIV‐uninfected pregnant women (9.1 [95% CI 7.5–10.8]; *p* < 0.01). We did not observe any significant associations between influenza rates when we compared those with and without any other underlying comorbidities excluding HIV (Table S3). Similarly, we did not find any significant differences in rates of influenza infection when we compared women who had malaria and those without malaria infection during pregnancy.

**TABLE 3A:** Incidence per 1,000 person‐months by HIV status, trimester during pregnancy and post‐partum period for mothers, June 2015 to May 2020

	Influenza—overall		Influenza among HIV (+)		Influenza among HIV (−)		Rate ratio	*p* value
Group	n/N (%)	aIR (95% CI)	n/N (%)	aIR (95% CI)	n/N (%)	aIR (95% CI)	(95% CI)	
Women								
All	170	7.9 (6.8, 9.1)	43	11.0 (7.9, 14.1)	127	7.2 (6.1, 8.5)	1.5 (1.1, 2.1)	0.01
Pregnancy	140 (82.4)	10.3 (8.6, 11.8)	38 (88.4)	15.6 (11.0, 20.6)	102 (80.3)	9.1 (7.5, 10.8)	1.7 (1.2, 2.5)	<0.01
Trimester 1	7 (5)	11.8 (4.8, 20.8)	3 (7.9)	30.4 (0.0, 69.6)	4 (3.9)	8 (1.9, 16.6)	4.3 (0.8, 23.3)	0.05
Trimester 2	62 (44.3)	9.0 (6.8, 11.2)	17 (44.7)	13.0 (7.1, 19.4)	45 (44.1)	8 (5.8, 10.4)	1.7 (0.9, 2.9)	0.07
Trimester 3	71 (50.7)	11.6 (9.1, 14.1)	18 (47.4)	16.7 (9.9, 23.9)	53 (52)	10.5 (7.9,13.5)	1.6 (0.9, 2.7)	0.07
Postpartum	30 (17.6)	4.0 (2.6, 5.5)	5 (11.6)	3.4 (0.7, 6.7)	25 (19.7)	4.1 (2.6, 5.6)	0.8 (0.3, 2.0)	0.72
Puerperium	10 (5.9)	3.3 (1.6, 5.3)	1 (2.3)	1.7 (0.0, 5.6)	9 (7.1)	3.5 (1.6, 6.0)	0.5 (0.0–3.4)	0.53
Maternal age								
15–19 years	15 (8.8)	5.9 (3.2, 8.9)	2 (4.7)	11.7 (0.0, 29.4)	13 (10.2)	5.5 (2.6, 8.8)	1.9 (0.2, 8.4)	0.39
20–33 years	140 (82.4)	8.3 (6.9, 9.7)	33 (76.7)	10.9 (7.5,14.8)	107 (84.3)	7.7 (6.3, 9.2)	1.4 (1.0, 2.0)	0.07
34–49 years	15 (8.8)	7.5 (4.2,11.7)	8 (18.6)	10.7 (4.1, 18.1)	7 (5.5)	5.6 (1.7, 10.3)	1.8 (0.6, 5.5)	0.22

Abbreviations: CI, confidence intervals; IR, incidence rate; HIV, human immunodeficiency virus.

### 3.6 ARI and influenza among infants

During follow‐up, a total of 2,042 ARI episodes occurred among 1,256 (49.3%) infants: 744 (59.2%) had a single ARI episode during follow‐up, 330 (26.3%) had two, and 182 (14.5%) had ≥3 ARIs (Table 2). Respiratory specimens were collected for 2,009 episodes, and 38 (2.0%) of 1,866 tested specimens were positive for influenza. Thirty‐two infants (1.3%) were hospitalized, 15 within 2 weeks of an ARI episode; all specimens tested negative for influenza.

### 3.7 Incidence rates of influenza among infants

The incidence of influenza per 1,000 person‐months among infants was 4.4 (95% CI 3.0–5.9) (5.4 [95% CI 3.1–8.1] per 100 person‐years) (Table 3B). The incidence was 8.7 (95% CI 4.8–12.9) among infants aged 3–6 months, more than double that among infants aged less than 3 months, which was 3.1 (95% CI 1.8–4.5); *p* < 0.01). Rates were similar among HIV‐exposed (4.3 [95% CI 1.3–7.6]) and unexposed infants (4.5 [95% CI 3.0–6.0]; *p* = 0.93). Among infants born to mothers with laboratory‐confirmed influenza during pregnancy, incidence of influenza (4.7 [95% CI 0.0–12.3]) was similar to incidence among infants born to mothers without documented influenza during pregnancy (4.4 [95% CI 3.0–5.8]; *p* = 0.98).

**TABLE 3B:** Incidence per 1,000 person‐months by HIV exposure, age in months and maternal influenza exposure for infants, June 2015 to May 2020

Group	Influenza–overall (*N* = 38)		Influenza among HIV exposed (*N* = 7)		Influenza among HIV unexposed (*N* = 31)		Rate ratio (95% CI)	*p* value
	*n* (%)	IR (95% CI)	*n* (%)	IR (95% CI)	*n* (%)	IR (95% CI)		
Infants								
All	38	4.4 (3.0, 5.9)	7	4.3 (1.3, 7.6)	31	4.5 (3.0, 6.0)	1.0 (0.4, 2.2)	0.93
0 to <3 months	21 (55.3)	3.1 (1.8, 4.5)	4 (57.1)	3.2 (0.8, 6.5)	17 (54.8)	3.1 (1.7, 4.8)	1.0 (0.2, 2.9)	0.99
3 to <6 months	17 (44.7)	8.7 (4.8, 12.9)	3 (42.9)	7.9 (0.0, 18.2)	14 (45.2)	8.9 (4.6, 13.9)	0.8 (0.1, 2.6)	0.70
Maternal influenza exposure								
Mother had influenza	2 (5.3)	4.7 (0.0, 12.3)	1 (14.3)	9.6 (0.0, 28.9)	1 (3.2)	3.1 (0.0, 10.0)	3.6 (0.0, 282.9)	0.43
Mother had no influenza	36 (94.7)	4.4 (3.0, 5.8)	6 (85.7)	4.0 (1.3, 7.9)	30 (96.8)	4.5 (3.0, 6.2)	0.9 (0.3, 2.1)	0.89

Abbreviations: CI, confidence intervals; IR, incidence rate; HIV, human immunodeficiency virus.

### 3.8 Influenza circulation

Influenza circulated during most months in the course of the study period with higher proportions of specimens testing positive for influenza between the months of March and April (7%–22%) and July and October (8%–20%) and peaked in March (11%) and June (22%) (Figure 1B). Influenza B predominated in 2015 and 2016, while influenza A/H3N2 dominated 2017 and 2019, and A/H1N1pdm09 dominated in 2018.

## 4 DISCUSSION

### 4.1 Main findings

During a 4‐year period, we identified a substantial burden of influenza‐associated respiratory illness among pregnant women and young infants in western Kenya. Incidence of laboratory‐confirmed influenza during pregnancy was twofold higher than during the postpartum period. Our study also found that HIV‐infected pregnant women had nearly twice the rate of laboratory‐confirmed influenza than HIV‐uninfected pregnant women. These findings suggest that whereas maternal vaccination may be beneficial for all women during pregnancy, vaccination may be especially important in reducing the burden of influenza among those who are HIV infected.

The maternal burden of influenza in Africa is under‐recognized because of lack of epidemiological and laboratory surveillance data.^31^ Moreover, relative to the general population, pregnant women are less likely to seek care for a respiratory illness,^32^, ^33^ and even when they do, influenza testing is not routinely done. In our study, we found that Kenyan pregnant women experience substantial morbidity from ARI compared with pregnant women in high‐income regions^4^, ^34^, ^35^ and other tropical upper middle‐income countries.^36^ We found influenza infection rates among pregnant women (10.3 [95% CI 8.6–11.8] per 1,000 person‐months) were similar to or greater than those reported in temperate regions (3.1 [95% CI 2.5–4.0] per 1,000 person‐months) and other tropical upper middle‐income regions (7.0–8.9 [95% CI 5.4–11.5] per 1,000 person‐months). Kenyan pregnant women were likely to experience higher morbidity associated with influenza illness, considering that influenza viruses are detected throughout the year. The burden was significantly higher among HIV‐infected pregnant women, consistent with findings from studies conducted in settings with high HIV prevalence.^37^, ^38^, ^39^ This underscores the need for maternal vaccination strategies to protect high‐risk groups. We observed significant morbidity associated with influenza infection during pregnancy compared with the postpartum period, consistent with previous studies that documented higher influenza and influenza‐associated disease burden during pregnancy than non‐pregnant periods.^6^, ^40^

Active surveillance for ARI episodes among infants aged <6 months also identified a substantial burden of influenza before these children are eligible to receive influenza vaccination. Incidence of laboratory‐confirmed influenza of 5.4 (95% CI 3.1–8.1) per 100 person‐years of follow‐up is comparable with rates of influenza illness observed among infants <6 months in countries that prioritize vaccination of mothers for the protection of their unborn infants, for example, in the United States which have ranged from 2.8 (95% CI: 0.7–11.1) to 5.9 (95% CI: 2.8–12.8) cases per 100 person‐years.^41^ Studies conducted in other LMICs have reported similar annual influenza incidence for infants younger than 6 months to those estimated in Kenya.^9^ Whereas we observed increased incidence in pregnant women with HIV, we did not identify increased incidence in HIV‐exposed infants, possibly due to small sample size. Even though we found no difference in risk of influenza among the two groups of infants, prioritizing influenza vaccine for women living with HIV is still a reasonable strategy for both the mother and baby, especially when resources are insufficient to vaccinate all pregnant women and/or their infants aged >6 months.

The incidence of influenza among infants aged 3–6 months in our study was twice that of infants aged <3 months. Our findings suggest that antibody transfer from mothers naturally infected with influenza during pregnancy may offer protection to infants in their early months of life.^42^ A similar response achieved through vaccinating pregnant women using inactivated influenza vaccines has been documented.^19^, ^38^, ^43^, ^44^, ^45^

### 4.2 Strengths of the study

Our study had several strengths. First, we employed a prospective cohort design with laboratory‐confirmed influenza as the outcome with molecular testing by an accredited laboratory. This enabled us to collect suitable data to describe influenza‐specific disease burden. Second, weekly surveillance for ARI increased our chances of detecting most episodes of illness and early enough to enable sampling within a window when PCR is most likely to identify viruses that are shed. Third, we had more than 3 years of surveillance data to assess the burden of influenza over years and examine year‐to‐year variability in the predominant influenza type/subtype and timing for circulating strains. Finally, this study maintained high retention rates and obtained respiratory specimens from nearly 90% of mothers and infants reporting symptoms of ARI.

### 4.3 Limitations of the study

The following limitations should be considered when interpreting our findings. First, because of the limited size of our cohort, we were unable to assess the impact of influenza on severe outcomes such as hospitalization and death. Second, it is possible that women were more motivated to report ARIs during pregnancy than immediately after birth especially if a report would lead to a clinic visit for a new mother with a neonate and potentially other children to attend to. These would potentially lead to underestimation of the burden among postpartum mothers. Similarly, the median postpartum person‐time contributed was significantly less compared with the intended 6‐month postpartum follow‐up. Finally, it is possible that HIV‐infected women were more motivated to report ARIs compared with those who were HIV‐uninfected, because they are educated on importance of early healthcare seeking for better treatment outcomes.

## 5 CONCLUSION

Our study shows a substantial burden of influenza‐associated illness during pregnancy, especially among HIV‐infected mothers and suggests the potential benefits of influenza vaccination during pregnancy to mothers and their infants. This evidence suggests that Kenyan authorities should explore the potential value of vaccinating pregnant women, especially those who are HIV infected to protect them and their unborn infants from influenza illness.

## AUTHOR CONTRIBUTIONS

**Nancy Otieno:** Conceptualization; data curation; formal analysis; funding acquisition; investigation; methodology; project administration; resources; supervision. **Bryan Nyawanda:** Data curation; formal analysis; validation. **Meredith McMorrow:** Conceptualization; data curation; funding acquisition; investigation; methodology. **Martina Oneko:** Data curation; investigation; project administration; supervision. **Daniel Omollo:** Data curation; investigation; project administration; supervision. **Shirley Lidechi:** Data curation; formal analysis; resources; supervision; validation. **Marc‐Alain Widdowson:** Conceptualization; funding acquisition; investigation; methodology; project administration; supervision. **Brendan Flannery:** Formal analysis; investigation; methodology. **Sandra S Chaves**: Conceptualization; funding acquisition; data curation; supervision; formal analysis; methodology. **Eduardo Azziz‐Baumgartner:** Formal analysis; funding acquisition; investigation; methodology. **Gideon Emukule:** Formal analysis; methodology; validation.

## CONFLICT OF INTEREST

The authors declare that they have no competing interests. Dr. SS Chaves has since left the Centers for Disease Control and Prevention (CDC) and is currently an employee of Sanofi Pasteur.

## FUNDING INFORMATION

This work was supported by funding from the U.S. Centers for Disease Control and Prevention, through the National Center for Immunization and Respiratory Diseases, Influenza Division (Grant number GH002133).

## ETHICAL APPROVAL

Ethical clearance for the study was obtained from KEMRI Scientific and Ethics Review Unit (KEMRI SSC. 2880) and CDC's institutional review board (CDC Protocol #6709) in February 2015. Informed consent was obtained from all participants before enrolment in the study.

## DISCLAIMER

The findings and conclusions in this article are those of the authors and do not necessarily represent the official position of the U.S. Centers for Disease Control and Prevention (CDC) or the Kenya Medical Research Institute (KEMRI).

### PEER REVIEW

The peer review history for this article is available at https://publons.com/publon/10.1111/irv.12950.

## Supporting information


**Table S1:** Pregnancy Outcomes in the Maternal–infant Study in Kenya, June 2015–May 2020, N = 3026
**Table S2:** Demographic and Clinical Characteristics of Pregnant Women Lost to Follow‐up Compared with Women who Completed the Maternal–infant Study in Kenya, June 2015–May 2020, N = 2972
**Table S3:** Incidence per 1000 Person‐months by Underlying Medical Condition (excluding HIV) and Trimester during Pregnancy and Post‐partum Period for Mothers, June 2015–May 2020
**Table S4:** Incidence per 1000 Person‐months by Year for Women and Infants, June 2015–May 2020

## Data Availability

The data that support the findings of this study are available from the corresponding author after permission from relevant authority to release data upon reasonable request.
