# Supplementary material for: The burden of influenza among Kenyan pregnant and postpartum women and their infants, 2015–2020
Source: Influenza Other Respir Viruses. 2022 Jan 23;16(3):452–61. doi: 10.1111/irv.12950 (PMC8983887; doi:10.1111/irv.12950)
Supplement: Supplementary file 1 — Table S1: Pregnancy Outcomes in the Maternal–infant Study in Kenya, June 2015–May 2020, N = 3026 Table S2: Demographic and Clinical Characteristics of Pregnant Women Lost to Follow‐up Compared with Women who Completed the Maternal–infant Study in Kenya, June 2015–May 2020, N = 2972 Table S3: Incidence per 1000 Person‐months by Underlying Medical Condition (excluding HIV) and Trimester during Pregnancy and Post‐partum Period for Mothers, June 2015–May 2020 Table S4: Incidence per 1000 Person‐months by Year for Women and Infants, June 2015–May 2020 [file IRV-16-452-s001.docx]

| Table S1: Pregnancy Outcomes in the Maternal-infant Study in Kenya, June 2015 - May 2020, N=3,026 | | | | |  |
| --- | --- | --- | --- | --- | --- |
| **Characteristic** | | **n** | | **%** |  |
| Miscarriages | 54 | | 1∙8 | | |
| Births |  | |  | | |
| Still birth | 55 | | 2∙1 | | |
| Live birth | 2550 | | 97∙9 | | |
| Place of delivery |  | |  | | |
| Study hospital | 2120 | | 83∙1 | | |
| Other health facility | 360 | | 14∙1 | | |
| Home | 70 | | 2∙8 | | |
| Mode of delivery |  | |  | | |
| C-section | 156 | | 6∙1 | | |
| Vaginal delivery | 2394 | | 93∙9 | | |
|  |  | |  | | |
| Complications during pregnancy n=2,708 |  | |  | | |
| Premature membrane rapture | 1 | | 0∙0 | | |
| Antepartum hemorrhage, (including abruption placentae and placenta previa) | 5 | | 0∙2 | | |
| Meconium –stained amniotic fluid | 10 | | 0∙4 | | |
| Hypertension in pregnancy (including eclampsia, preeclampsia and pre-eclampsia toxemia) | 11 | | 0∙4 | | |
|  |  | |  | | |
| Maternal death | 5 | | 0∙2 | | |
| Postpartum | 5 | | 100∙0 | | |
| <14 days | 3 | | 60∙0 | | |
| 14–28 days | 1 | | 20∙0 | | |
| 29 days–12 weeks | 1 | | 20∙0 | | |
| Cause of death^†^ |  | |  | | |
| HIV related complications | 2 | | 40∙0 | | |
| Postpartum hemorrhage | 2 | | 40∙0 | | |

^†^One death occurred at home and cause of death could not be established.

Abbreviation: HIV, human immunodeficiency virus.

Table S2: Demographic and Clinical Characteristics of Pregnant Women Lost to Follow-up Compared with Women who Completed the Maternal-infant Study in Kenya, June 2015 - May 2020, N=2972.

| **Characteristic** | **Lost to follow-up** | | **Completed the study** | | ***p*-value** |  |
| --- | --- | --- | --- | --- | --- | --- |
| Age at enrolment |  |  | |  | | |
| <25 | 233 (63∙49) | 1353 (51∙94) | | <0∙01 | | |
| 25 - 34 | 119 (32∙43) | 1091 (41∙88) | |  | | |
| >=35 | 15 (4∙09) | 161 (6∙18) | |  | | |
|  |  |  | |  | | |
| First pregnancies | 147 (40∙05) | 646 (24∙80) | | <0∙01 | | |
| First trimester | 107 (29∙16) | 737 (28∙29) | | 0∙54 | | |
| Second trimester | 260 (70∙84) | 1860 (71∙40) | |  | | |
| Third trimester | 0 (0.0) | 8 (0∙31) | |  | | |
|  |  |  | |  | | |
| Chronic condition | 15 (4∙09) | 101 (3∙88) | | 0∙85 | | |
| Asthma | 9 (2∙45) | 64 (2∙46) | | 0∙99 | | |
| COPD | 0 (0.0) | 4 (0∙15) | | 0∙20 | | |
| HIV | 48 (13∙08) | 493 (18∙93) | | 0∙01 | | |
| TB or on TB treatment | 3 (0∙82) | 11 (0∙42) | | 0∙47 | | |
| Diabetes | 0 (0.0) | 6 (23) | | 0∙61 | | |
| Hypertension | 8 (0∙82) | 14 (0∙54) | | 0∙75 | | |
| Epilepsy | 0 (0.0) | 8 (0∙31) | | 0∙49 | | |
|  |  |  | |  | | |

Abbreviations: COPD, chronic obstructive pulmonary disease; HIV, human immunodeficiency virus;

TB, tuberculosis

| Table S3: Incidence per 1,000 Person-months by Underlying Medical Condition (excluding HIV) and  Trimester during Pregnancy and Post-partum Period for Mothers, June 2015–May 2020. |
| --- |

| Group | | Underlying medical conditions^†^ | | | | No underlying medical conditions | | | | | Rate Ratio  (95% CI) | | *p*-value | | | |
| --- | --- | --- | --- | --- | --- | --- | --- | --- | --- | --- | --- | --- | --- | --- | --- | --- |
|  | | **n/N (%)** | | **aIR (95% CI)** | | | **n/N (%)** | | **aIR (95% CI)** | | |  | | |  | |
| All | 5 | | 6∙0 (1∙2,11∙5) | | 165 | | | 8∙0 (6∙8,9∙2) | | 0∙8 (0∙3,1∙7) | | | | 0∙59 | |  |
| Pregnancy | 4 (80∙0) | | 7∙3 (1∙8,15∙6) | | 136 (82∙4) | | | 10∙3 (8∙7,12∙1) | | 0∙6 (0∙2,1∙7) | | | | 0∙39 | |  |
| Trimester 1 | 0 (0∙0) | | 0∙0 (0∙0,0∙0) | | 7 (5∙1) | | | 10∙5 (4∙5,17∙9) | | 0∙0 (0∙0,21∙3) | | | | 0∙80 | |  |
| Trimester 2 | 3 (75∙0) | | 10∙6 (0∙0,24∙7) | | 59 (43∙4) | | | 7∙9 (6∙1,9∙8) | | 1∙2 (0∙2,3∙7) | | | | 0∙70 | |  |
| Trimester 3 | 1 (25∙0) | | 4∙0 (0∙0,13∙3) | | 70 (51∙5) | | | 10∙4 (8∙0,12∙8) | | 0∙4 (0∙0,2∙0) | | | | 0∙30 | |  |
| Post-partum | 1 (20∙0) | | 3∙2 (0∙0,11∙3) | | 29 (17∙6) | | | 3∙9 (2∙7,5∙4) | | 0∙7 (0∙0,4∙4) | | | | 0∙86 | |  |
| Puerperium | 1 (20∙0) | | 8∙1 (0∙0,25∙8) | | 9 (5∙5) | | | 3∙0 (1∙3,5∙3) | | 2∙8 (0∙1,19∙9) | | | | 0∙38 | |  |

^†^Underlying medical conditions included Asthma, chronic obstructive pulmonary disease, bronchitis, tuberculosis, diabetes, hypertension and epilepsy.

Abbreviations: CI, confidence intervals; IR, incidence rate; HIV, human immunodeficiency virus.

Table S4: Incidence per 1,000 Person-months by Year for Women and Infants, June 2015–May 2020.

Abbreviations: CI, confidence intervals; IR, incidence rate; HIV, human immunodeficiency virus.

| **Year** | **Influenza – Overall** | | | **Influenza among HIV (+)** | | | | **Influenza among HIV (-)** | | **Rate Ratio** |  | |
| --- | --- | --- | --- | --- | --- | --- | --- | --- | --- | --- | --- | --- |
|  | **n/N (%)** | **IR (95% CI)** | | **n/N (%)** | **IR (95% CI)** | | | **n/N (%)** | **IR (95% CI)** | **(95% CI)** | ***p*-value** | |
| Women | N=170 | |  | N=43 | |  | N=127 | |  |  |  |  |
| All years | 170 | 8∙0 (6∙8, 9∙1) | | 43 | 10∙9 (7∙9, 14∙4) | | 127 | | 7∙2 (6∙1, 8∙5) | 1∙5 (1∙1, 2∙1) | 0∙01 |  |
| Jun 2015–May 2016 | 27 (15∙9) | 11∙0 (7∙4, 15∙2) | | 7 (16∙3) | 19∙0 (5∙7, 33∙6) | | 20 (15∙7) | | 9∙6 (5∙8, 13∙8) | 1∙9 (0∙7, 4∙5) | 0∙17 |  |
| Jun 2016–May 2017 | 38 (22∙4) | 6∙0 (4∙2, 8∙0) | | 10 (23∙3) | 8∙5 (4∙2, 13∙8) | | 28 (22) | | 5∙4 (3∙7, 7∙5) | 1∙6 (0∙7, 3∙2) | 0∙18 |  |
| Jun 2017–May 2018 | 58 (34∙1) | 12∙6 (9∙6, 15∙7) | | 15 (34∙9) | 17∙0 (9∙0, 25∙3) | | 43 (33∙9) | | 11∙7 (8∙6, 15) | 1∙4 (0∙8, 2∙5) | 0∙21 |  |
| Jun 2018–May 2019 | 21 (12∙4) | 3∙9 (2∙3, 5∙7) | | 7 (16∙3) | 7∙2 (2∙2, 12∙5) | | 14 (11) | | 3∙1 (1∙6, 4∙9) | 2∙5 (0∙9, 6∙2) | 0∙05 |  |
| Jun 2019–May 2020 | 26 (15∙3) | 9∙5 (6∙1, 13∙3) | | 4 (9∙3) | 7∙1 (1∙7, 14∙8) | | 22 (17∙3) | | 9∙9 (6, 14∙6) | 0∙7 (0∙2, 1∙9) | 0∙54 |  |
|  |  |  | |  |  | |  | |  |  |  |  |
| Infants | N=38 |  | | N=7 |  | | N=31 | |  |  |  |  |
| All years | 38 | 4∙4 (3∙0, 6∙0) | | 7 | 4∙3 (1∙2, 7∙6) | | 31 | | 4∙4 (2∙9, 6∙1) | 1∙0 (0∙4, 2∙2) | 0∙93 |  |
| Jun 2015–May 2016 | 1 (2∙6) | 2∙0 (0∙0, 8∙0) | | 0 (0) | 0 (0, 0) | | 1 (3∙2) | | 2∙3 (0∙0, 7∙2) | 0∙0 (0∙0, 240∙9) | 0∙86 |  |
| Jun 2016–May 2017 | 2 (5∙3) | 1∙0 (0∙0, 2∙5) | | 0 (0) | 0 (0, 0) | | 2 (6∙5) | | 1∙2 (0∙0, 3∙0) | 0∙0 (0∙0, 22∙5) | 0∙65 |  |
| Jun 2017–May 2018 | 11 (28∙9) | 6∙6 (3∙0, 10∙9) | | 3 (42∙9) | 9∙4 (0∙0, 22∙2) | | 8 (25∙8) | | 6∙0 (2∙3, 10∙6) | 1∙5 (0∙3, 6∙3) | 0∙54 |  |
| Jun 2018–May 2019 | 9 (23∙7) | 3∙6 (1∙6, 6∙4) | | 1 (14∙3) | 2∙3 (0∙0, 7∙1) | | 8 (25∙8) | | 3∙9 (1∙5, 6∙8) | 0∙6 (0∙0, 4∙3) | 0∙68 |  |
| Jun 2019–May 2020 | 15 (39∙5) | 8∙1 (3∙9, 12∙8) | | 3 (42∙9) | 7∙7 (0∙0, 17∙7) | | 12 (38∙7) | | 7∙9 (4∙0, 13) | 1∙0 (0∙2, 3∙2) | 0∙94 |  |
